# Interview With Barbara Pearse and the Clathrin Family Tree

**DOI:** 10.1111/tra.70048

**Published:** 2026-08-27

**Authors:** Frances M. Brodsky, Margaret S. Robinson

**Affiliations:** ^1^ Research Department of Structural and Molecular Biology Division of Biosciences, University College London London UK; ^2^ Cambridge Institute for Medical Research Keith Peters Building University of Cambridge Cambridge UK

## Abstract

The clathrin family tree. Timeline of research on clathrin leading to and generated by Barbara Pearse's discovery, with milestones mapped onto the clathrin triskelion.

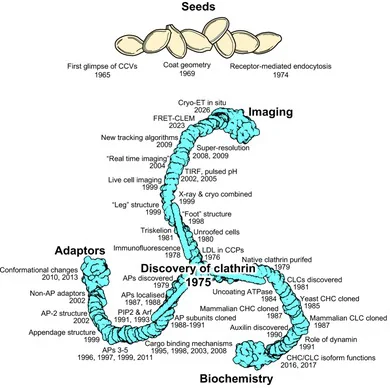

It was a dark and stormy day in Cambridge in January 2026 when we, Margaret (Scottie) Robinson and Frances Brodsky, set out to visit Barbara Pearse and asked her to reminisce about her discovery of the clathrin protein, 50 years earlier. Scottie and Frances have spent the better part of our scientific careers studying clathrin‐mediated membrane traffic, defining its molecular mechanisms and physiological functions. Barbara influenced both of us early in these careers. Scottie joined Barbara's laboratory in 1982 at the Medical Research Council Laboratory of Molecular Biology in Cambridge, UK, as a postdoctoral fellow funded by the US National Institutes of Health, and started her fellowship by characterizing clathrin adaptors [1]. Frances had just completed her Damon Runyon postdoctoral fellowship at Stanford University, USA, in Peter Parham's laboratory, where she met Barbara who was on a sabbatical visit to Nigel Unwin's lab next door. Barbara shared her clathrin purification protocol with Frances, greatly enabling the second generation of monoclonal antibodies against clathrin that Frances produced [2] following her initial collaboration with Tomas (Tommy) Kirchhausen and Stephen Harrison at Harvard University [3]. Our work with clathrin was part of a growing interest in molecular aspects of membrane traffic and clathrin that had simultaneously caught the interest of a number of cell and structural biologists. Barbara's identification in 1975 of clathrin as the major protein in brain coated vesicles [4], and her demonstration that clathrin was the major component of similar coated vesicles from several other tissues in 1976 [5], was an essential contribution to this nascent interest in the field. Since then, Barbara's work and that of others burgeoned into the clathrin family tree (Figure 1) that has defined clathrin‐mediated membrane traffic as we understand it today.

**FIGURE 1:**
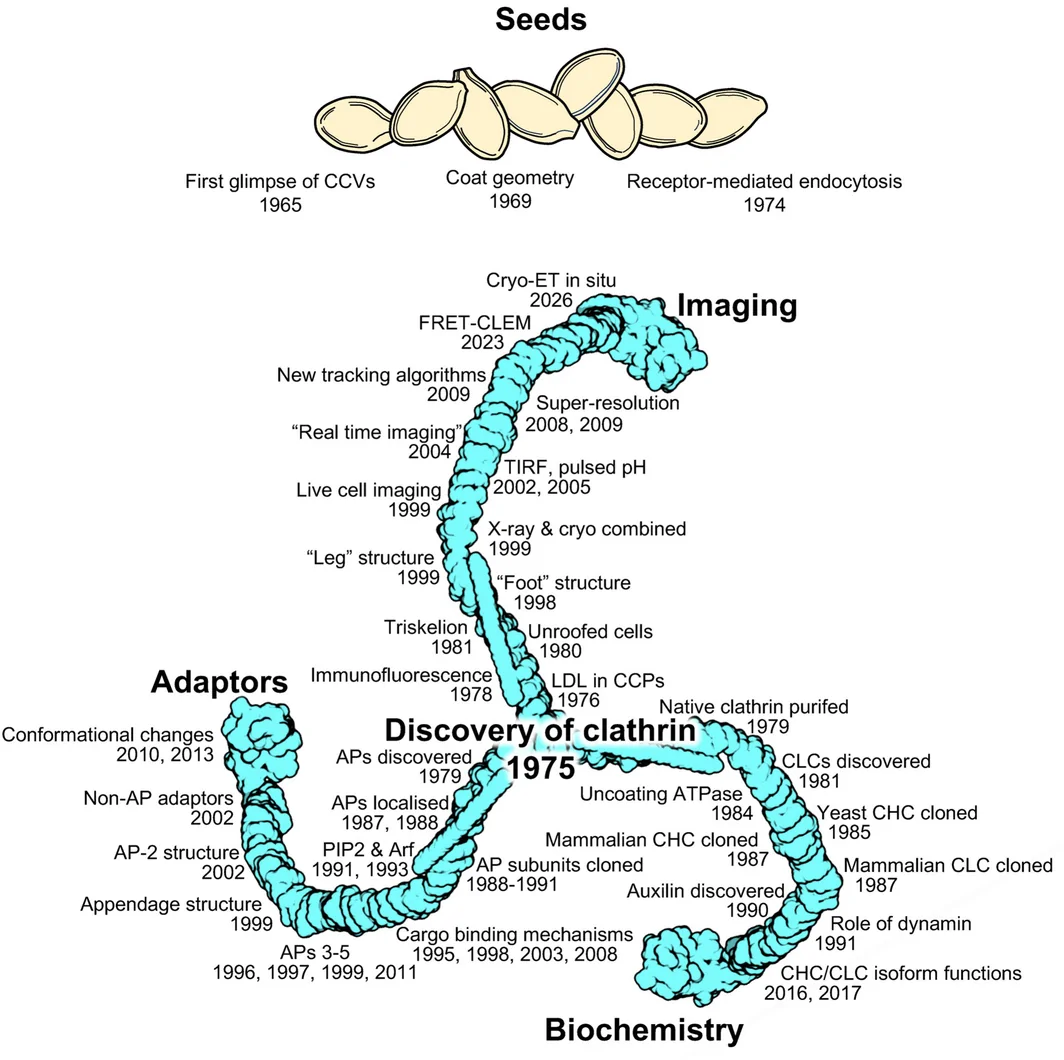
The clathrin family tree. Shown are the seeds for Barbara Pearse's discovery of clathrin and the areas of research on clathrin structure and function that her discovery generated. This figure summarizes many landmarks in the field with primary references listed below. Sincere apologies to colleagues for inevitable omissions. Clathrin research is still flourishing and welcomes new investigators with new technologies that continue to reveal remarkable features and functions of clathrin proteins. The triskelion image is reproduced and the angle slightly altered from the illustration in PDB‐101 Molecule of the Month entry by Graham T. Johnson and David S. Goodsell http://doi.org/10.2210/rcsb_pdb/mom_2007_4availableunderaCC‐BY‐4.0license. Seeds: First glimpse of CCVs, 1964 [6]. Coat geometry, 1969 [7]. Receptor‐mediated endocytosis, 1974 [8]. Imaging: LDL (low density lipoprotein receptor) in CCPs (clathrin‐coated pits), 1976 [9]. Immunofluorescence, 1978 [10]. Unroofed cells, 1980 [11]. Triskelion, 1981 [12, 13]. “Foot” (N‐terminal domain) structure, 1998 [14]. “Leg” (proximal region) structure, 1999 [15]. Cryo EM (electron microscopy) 1998 [16]. Live cell imaging; 1999 [17]. TIRF (total internal reflection microscopy), pulsed pH, 2002, 2003, 2005 [18, 19, 20]. “Real time imaging,” 2004 [21]. Cryo EM & Xray combined, 2004 [22]. Super‐resolution, 2008, 2009 [23, 24]. New tracking algorithms, 2009 [25]. Cryo‐ET (electron tomography) in situ, 2012, 2021, 2026 [26, 27, 28]. FRET (fluorescence resonance energy transfer)‐CLEM (correlative light and EM), 2023 [29]. Biochemistry: Native clathrin purified, 1979 [30]. CLCs (clathrin light chains) discovered, 1981 [12, 13]. Uncoating ATPase, 1984 [31]. Yeast CHC (clathrin heavy chain) cloned, 1985 [32]. Mammalian CHC cloned, 1987 [33]. Mammalian CLCs cloned, 1987 [34, 35]. Auxilin discovered, 1990 [36]. Role of dynamin discovered, 1991 [37, 38]. CHC at the mitotic spindle, 2005 [39]. CHC/CLC isoform functions, 2009, 2016, 2017, 2019 [40, 41, 42, 43]. CLC role in clathrin assembly, 2010 [44]. Clathrin roles in Arabadopsis, 2011 [45]. Novel CLC in Giardia, 2022 [46]. Adaptors: APs (adaptor protein complexes) discovered, 1979 [30]. APs localized, 1987, 1988 [47, 48]. AP subunits cloned, 1988–1991 [49, 50, 51, 52, 53]. PIP2 (phosphotidyl inositol phosphate‐2) & ARF (ADP‐ribosylation factor) binding, 1991, 1993 [54, 55]. Arrestin binding to AP2 (1996) [56]. Cargo binding mechanisms, 1995, 1998, 2003, 2008 [57, 58, 59, 60]. APs 3–5, 1996, 1997, 1999, 2011 [61, 62, 63, 64, 65, 66]. Appendage structures, 1999, 2002 [67, 68]. AP‐2 structure, 2002 [69]. Non‐AP adaptors, 2002 [70, 71]. Conformational changes, 2010, 2013 [72, 73]. T‐PLATE adaptor, 2014 [74, 75].

Our pilgrimage to the cottage (Figure 2) where Barbara Pearse and her husband Mark Bretscher, also a well‐known molecular cell biologist, live at Swaffham Bulbeck was challenged by the wet roads, and that we were rather damp having been caught in the winter rain prior to setting off, and that both of us had learned to drive in the USA. Nonetheless, we arrived in time for tea and found Barbara willing to reminisce about her ground‐breaking discovery at a time when proteins responsible for intracellular processes were just beginning to be identified. Our conversation (essentially a random set of memories from Barbara, Mark and both of us, punctuated by a few questions) is paraphrased below. We note that an excellent and very thorough interview of both Barbara and Scottie covering the early days of clathrin research was published in 2019 in “Ahead of the Curve: women scientists at the MRC Laboratory of Molecular Biology” (LMB) by Kathleen Weston [76]. Additionally, Barbara Pearse's “EMBO medal review: Clathrin and coated vesicles” contains several historical anecdotes as well as the evolution of concepts of receptor sorting and membrane transport arising simultaneously that were fueled by her discovery of clathrin [77]. For those unfamiliar with that early history, we cover some of it in our present interview and were able also to address some further historical details.

**FIGURE 2:**
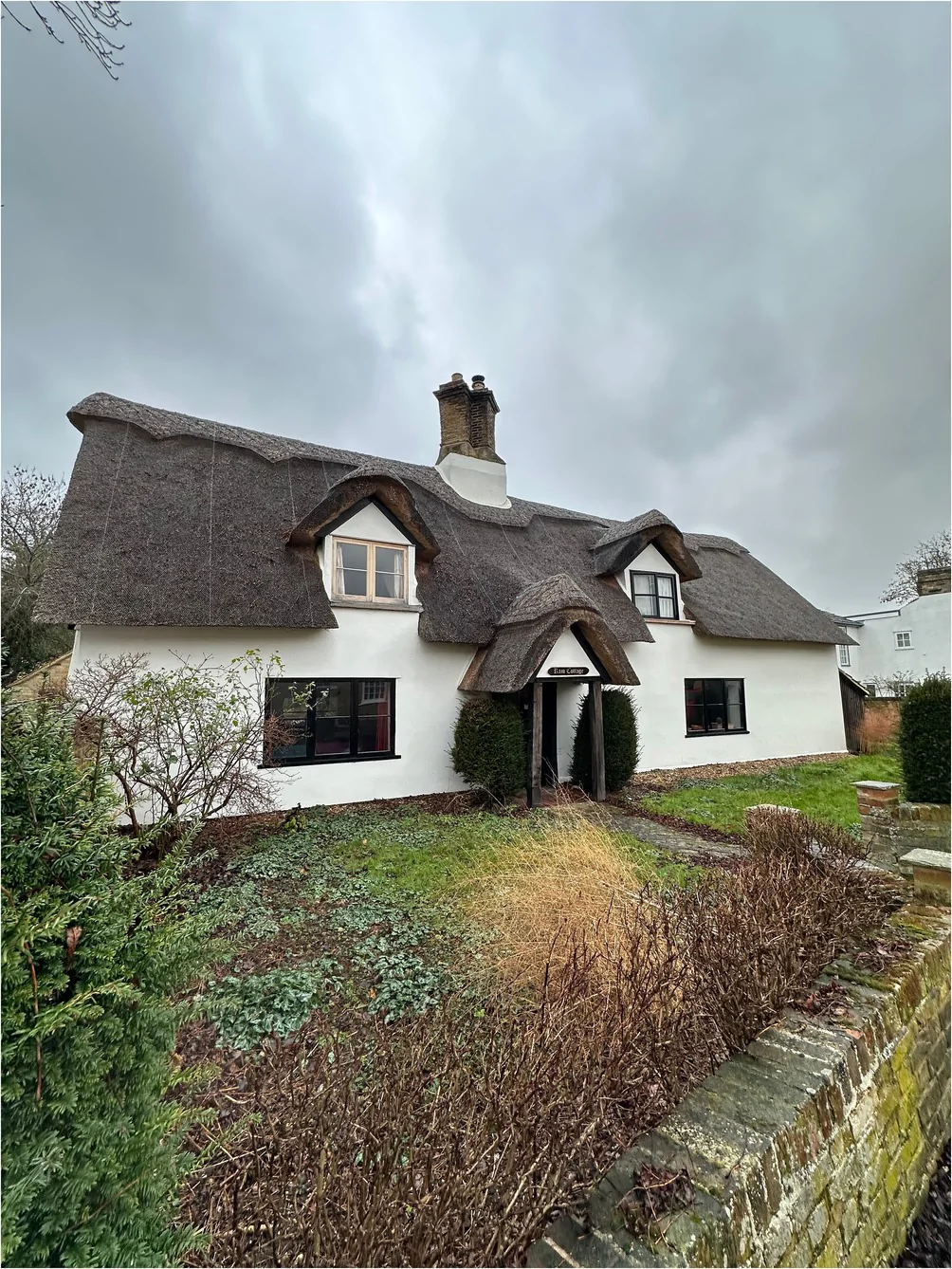
The cottage at Swaffham Bulbeck.

*We started by asking Barbara to remind us how her discovery of clathrin arose from identification of a “contaminant” during her attempted purification of microtubules.*

Barbara quickly corrected us to say that clathrin‐coated vesicles (CCVs) were not so much a contaminant but constituted the only visible product of an early attempt to purify microtubules. The purification had been scuppered by provision of porcine brains stored at 4°C overnight by the unwitting local butchers, who did not understand the meaning of “fresh” tissue. Of course, the cold temperature had depolymerized the microtubules, leading to no yield of tubulin but a residual pellet of structures that Barbara decided to “have a look at anyway” by electron microscopy out of frustrated curiosity. Upon seeing CCVs, which to her eyes resembled “sliced tomatoes,” Barbara approached colleagues at the LMB to ask what they thought these structures might be. Colleagues Dennis Bray and Clark Slater, also nascent cell biologists, pointed her to a 1969 paper by Kanaseki and Kadota, who had identified CCVs amongst purified guinea pig brain synaptosomes, and described them as a “vesicle in a basket” [7]. These, in turn, had been speculated to be the coated vesicles that Roth and Porter had described in 1964 in mosquito oocytes during uptake of yolk protein [6], thus already associated with their potential role in membrane traffic, coincidentally a field that Mark Bretscher had begun to take an interest in [78]. In her 1975 paper, Barbara identified that the major component of these vesicles was a “massive” (her word) band of 180 kilodaltons, that she proposed to call clathrin [4]. Barbara reminded us that the name was suggested by Graeme Mitchison, another colleague at the LMB, apparently the resident Greek scholar whom she consulted at Mark's suggestion. Clathrin derives from the Latin *clathrus*, meaning lattice, evolving from the Greek *kleithron*, meaning lattice‐work. At the time Mitchison also suggested two other names, that neither Barbara nor Mark could recall, with Barbara pointing out that clathrin was indeed the most “snappy,” which is why it was adopted. Our conversation later in the interview returned to the important role of the abattoir in our work. Barbara initially patronized a pork butcher in Cherry Hinton, then adjusted to the use of bullock brains from a butcher in Bury St Edmonds when Scottie was in the laboratory, given they could get greater quantities of tissue. Scottie recalled the gruesome sight of hanging bullock heads from which the brains were removed by cleaver and, of course, that was before BSE when UK labs returned to studying porcine clathrin. Frances noted that the abattoir in San Jose, California, would only distribute samples on their doorstep, but were so obliging in providing different fresh organs for analysis of clathrin light chain tissue polymorphism (incidentally prefigured by Pearse in her 1976 [5] and 1978 [79] papers) that Frances acknowledged the abattoir's contribution in one of her manuscripts [80]. Barbara and Mark also recounted shared acquisition of a whole calf with Roger Kornberg, who needed very fresh thymus for his studies of chromatin, that they made the most of by roasting the remains in the back garden at Swaffham Bulbeck after the relevant tissues were harvested for lab work.

*We next addressed how Barbara's discovery of clathrin was received by the scientific world—whether it was a competitive environment or a welcoming one in the late 1970's.*

According to both Barbara and Mark, her discovery was enthusiastically embraced and promoted by George Palade, who had also observed vesicles with evident protein coats in electron micrographs depicting intracellular structures. Barbara's 1976 paper describing clathrin as a major component of coated vesicles purified from adrenal tissue and lymphoid cells, as well as from brain [5], was communicated in January of that year to the Proceedings of the National Academy of Sciences (USA) by Fred Sanger and handled editorially by one of the George Palade‐Marilyn Farquhar cell biology duo. Apparently (and remarkably), around the same time, the other received a submission from Michael (Mike) Brown and Joseph (Joe) Goldstein who had teamed up with electron microscopist Richard (Dick) Anderson to study the localization of mutant LDL receptors [9]. The latter paper linked coated membrane regions of the plasma membrane to areas of selective endocytic receptor‐cargo uptake and the former demonstrated that the coats that might mediate this type of membrane traffic were formed from clathrin. Barbara recalls that this synchrony of ideas was received well by the field; the reaction was “oh wow” (in her words), as it introduced biochemical analysis to membrane traffic pathways. Conveniently, Mark Bretscher had embarked on an exploration of the role of lipid exchange and flow in cell membranes and had been thinking separately about the dynamics of endocytosis, further inspiring Barbara to continue with her studies of clathrin and its function, and also leading to several collaborative papers from the two of them about mechanisms of membrane traffic [81, 82]. These joint interests prompted their arranged sabbatical year at Stanford University in 1984–1985 with Nigel Unwin and Jim Rothman, with Barbara exploring the co‐assembly of mannose‐6‐phosphate receptors with clathrin coats and Mark developing his ideas about membrane flow and endocytosis. Back in Cambridge that year, Scottie was working in Barbara's lab, separating and characterizing adaptor complexes [1, 47], while Barbara's advice helped Frances in California produce new monoclonal antibodies against the clathrin heavy chain that enabled identification of domains involved in clathrin assembly [83].

*To fill in some of the history between 1975 and the Stanford sabbatical year in 1985, we asked Barbara about her association with Ernst Ungewickell, whose pioneering work with Dan Branton uncovered the triskelion shape of the clathrin trimer* [13].

With Barbara's discovery of clathrin opening up a biochemical angle on membrane traffic, other biochemists and microscopists were immediately drawn to study the protein. These included the team of James (Jim) Keen and Ira Pastan, who characterized self‐assembly of purified clathrin in 1979 and the participation of the adaptors that were separated from clathrin by gel filtration [30], as well as the morphologist John Heuser whose unique micrographs visualizing unroofed cells revealed the diversity of clathrin assemblies at the plasma membrane (1980) [11]. Scottie herself had been attracted to clathrin by Barbara's early publications and spoke with Dan Branton about the protein in the context of her role as a graduate teaching assistant in his Harvard cell biology course during the early years of her PhD. Branton, whose expertise was in characterizing the biochemistry of red blood cell cytoskeletal and membrane proteins, informed Scottie that one of his postdoctoral fellows, Ernst Ungewickell, was beginning to study clathrin. At the same time, also at Harvard, Tommy Kirchhausen had joined Stephen Harrison's lab to characterize coated vesicles using approaches that Steve's lab had pioneered in his analysis of polyhedral viral coats. Scottie herself eventually turned to a different project for her PhD work, saving her clathrin fascination for postdoctoral research. Meanwhile, both Ernst [13] and Tommy [12] had, through distinct approaches, arrived at clathrin's triskelion shape and predicted light chain subunit composition, as had Barbara, in pursuit of two hypotheses she put forward in 1976. With computational and structural biologists R. Anthony (Tony) Crowther and John T. Finch Barbara had suggested that clathrin was likely a trimer due to the three‐fold symmetry exhibited by different morphologies of clathrin lattices [84], and also in her 1976 and 1978 papers Barbara noted the persistent co‐purification of the clathrin light chain subunits [5, 79]. Not long after this further frenzy of clathrin characterization, Ernst Ungewickell spent a year in Barbara's lab, in transition to a post in Goettingen. His goal, Barbara said, was “to improve the clathrin prep”, which he achieved and passed on to Scottie upon her arrival in the laboratory, as well as informing her where to find the local ultracentrifuges. Ernst initially contributed the idea of using hydroxyapatite to separate adaptor complexes from each other, and along with Scottie, he and others launched the field of characterizing the function of these and other clathrin‐associated proteins that are essential components of CCVs [1, 85]. Meanwhile, Frances, enabled by Barbara's biochemical advice, continued on her own to use monoclonal antibodies to understand clathrin structure and assembly [83, 86]. The Kirchhausen–Harrison team, as well as Jim Keen, and then many others (Figure 1) also continued to make key contributions to structure–function analysis of clathrin‐coated vesicles and associated pathways.

*Having explored the impact of Barbara's discovery of clathrin in cell biology, we asked Barbara about her experience as a woman in science and how it affected her career.*

Barbara replied that she had always expected to get her PhD and have a career in science. As far back as she could remember, her physicist father, Reginald Pearse based at Imperial College, used to promise her that “when you've got your PhD, life will start.” Thus, Barbara enrolling for a Biochemistry undergraduate degree at UCL seemed a logical step toward this goal, even though UCL was a “radical” institution in her father's eyes. UCL was one of only two universities offering a degree in Biochemistry, which was the closest subject Barbara could find to address her interest in molecular biology, and she did not take to her interview for studying biochemistry at Oxford. Impressively, about a third of her degree class at UCL were young women (Figure 3), reflecting UCL's forward‐looking policy of awarding university degrees to women since its founding 200 years ago [87]. Barbara then joined Michael Rosemeyer's laboratory at UCL for her PhD research, which involved purification and characterization of the enzyme 6‐phosphogluconate dehydrogenase from human erythrocytes [88]. Rosemeyer's lab focused on enzymes critical for metabolism and when Barbara joined, a senior colleague in the lab finishing his PhD work was Philip Cohen, later known for his key contributions to understanding protein phosphorylation and oversight of the MRC Protein Phosphorylation and Ubiquitinylation Unit in Dundee. Barbara was then accepted for postdoctoral research by Ieuan Harris, based at the LMB in Cambridge to work on enzyme purification from thermophilic bacteria. Through her colleague Linda Amos at the LMB, who was analyzing tubulin by electron microscopy, Barbara became interested in the possibility of pursuing the biochemistry of tubulin, leading to the fortuitous isolation of CCVs and discovery of clathrin. In addition to Linda, there were a few other female colleagues at the LMB, but at that time no women group leaders. When Ieuan Harris died unexpectedly, Barbara obtained her own funding from a Beit Memorial Medical Fellowship to independently pursue her studies of clathrin in another department and finally cemented a tenured place to have her own group at the LMB in a third department, the Cell Biology Division, as her career matured. It likely helped that the Beit Fellowship was the most prestigious medical research fellowship at the time, which funded investigators who eventually received a total of seven Nobel Prizes (awardee Fred Sanger earned two) and also funded biochemist Majory Stephenson, one of the two first female fellows elected to the Royal Society in 1945 (the other was crystallographer Kathleen Lonsdale, the first female Professor at UCL). This Beit fellowship became the Wellcome‐Beit Prize fellowship in 2009, now folded into Wellcome early career awards. Notably, Albert Beit, memorialized by this fellowship established by his financier brother Otto Beit, was also a financier (railways) and philanthropist and established a still existing fund supporting researchers and entrepreneurs in Zimbabwe, Zambia and Malawi, as well as constructed the Otto Beit Bridge over the Zambezi River.

**FIGURE 3:**
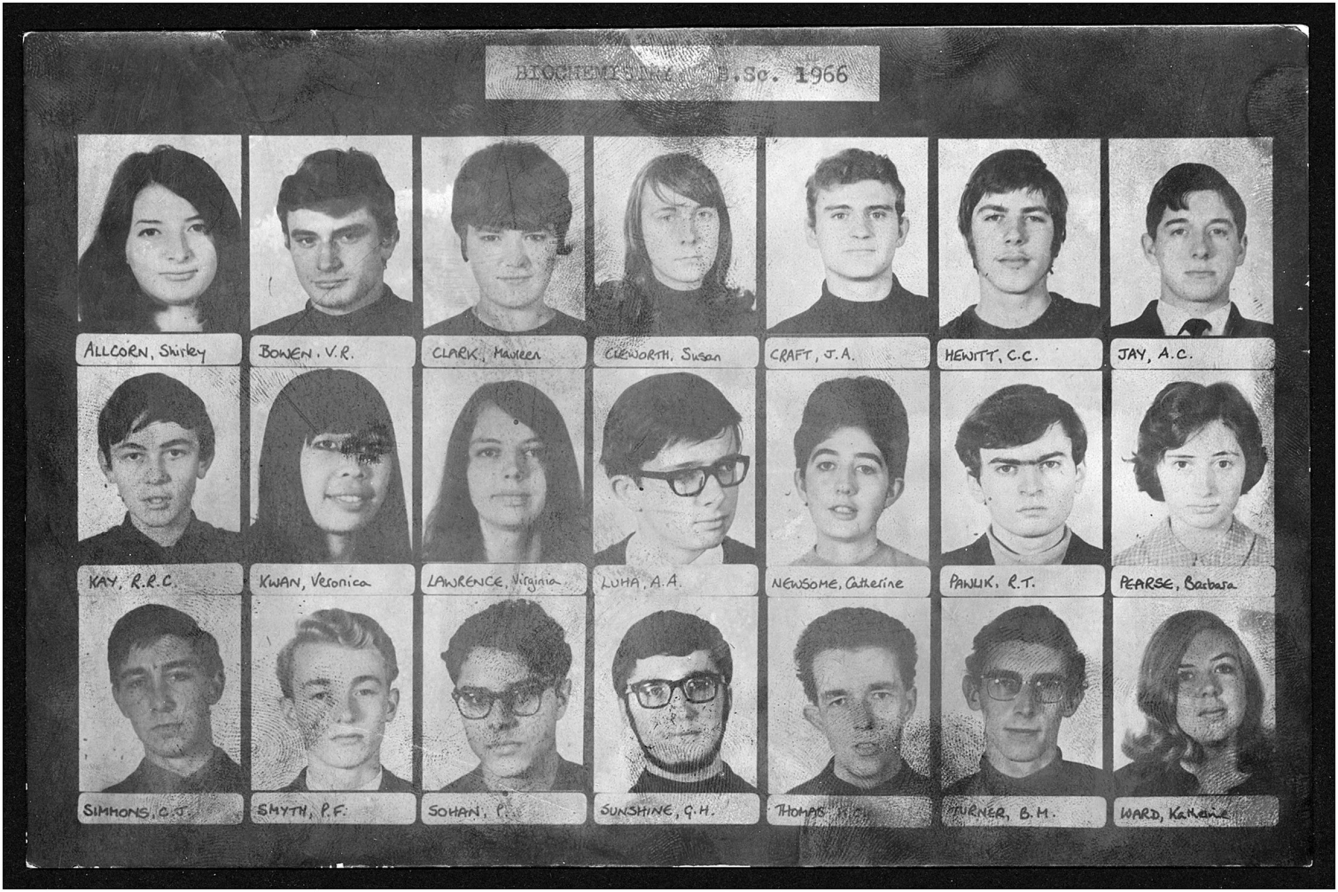
Undergraduates in the Biochemistry degree program at University College London, 1966 intake. Provided by Mark Bretscher.

Barbara was therefore an unusual beneficiary of visionary attitudes toward women who achieved scientific recognition. For her entire career, she ran a small laboratory at the LMB, continuing to work at the bench herself. Her research output from 1975 to the present listed by PubMed is less than 40 publications, virtually all of which contribute an advance to understanding clathrin structure and function. After characterizing clathrin biochemically, she pursued the important question of how clathrin coats act as molecular filters and interact with adaptors that recognize membrane‐embedded cargo and recruit clathrin lattices to membranes [77, 89]. She and Mark Bretscher predicted the existence of these functional complexes, pre‐naming them adaptors, a term (we were assured) that was purely coincidental with the earlier adaptor concept invented at LMB referring to tRNA. With the partnership of Scottie, as a postdoctoral fellow and then as an independent researcher in Cambridge, they defined fundamental aspects of adaptor composition and localization [47, 90]. Following on her studies of clathrin‐adaptor‐receptor interactions, Barbara capitalized on the cryo‐electron microscopy developed at the LMB, interacting and publishing with Guy Vigers [91]. An early finding we discussed was Peter Sorger's pursuit of clathrin cubes when he joined the LMB as a PhD student [92]. Barbara had observed these anomalies when assembling purified clathrin and Peter was determined to demonstrate their active production, if artificial existence. When electron microscopist Corinne Smith joined Barbara's lab as a postdoc, she and Barbara produced the first “high resolution” (21 Å) structural view of assembled clathrin that revealed how the triskelion legs intertwine to form a polyhedral lattice [16]. Frances recalls how exciting it was to receive this manuscript to review and gain this detailed insight into the clathrin assembly mechanism. Barbara welcomed young investigators who wanted to work with her. While not actively considering herself a scientific role model, she profoundly impacted the research careers of several women who came to work with her, as well as others she came into contact with. We have already mentioned ourselves (Scottie and Frances). Corinne Smith is now a Professor at the University of Warwick, who has continued to make seminal structural contributions to understanding clathrin biochemistry, assembly and uncoating, including her recent publication of the cryo‐EM structure of clathrin and adaptor fragments at 8–12 Å resolution [93]. Elizabeth (Liz) Conibear, now a Professor at the University of British Columbia, was a PhD student in Barbara's lab during Barbara's investigation of receptor‐adaptor‐clathrin interactions with Jonathan Glickman [94]. Liz continues to work in the field of membrane traffic, using yeast genetics to uncover novel regulatory proteins [95].

*Barbara Pearse's influence, of course, reached far beyond those who immediately worked with her. We closed the interview with tea and cake and a requisite selfie of the four of us by the fireplace in the Swaffham Bulbeck cottage* (Figure 4). *The story of clathrin's discovery and the new directions it spawned is one that proves the value of serendipity and generosity in science and set the stage for future generations of molecular cell biologists.*

**FIGURE 4:**
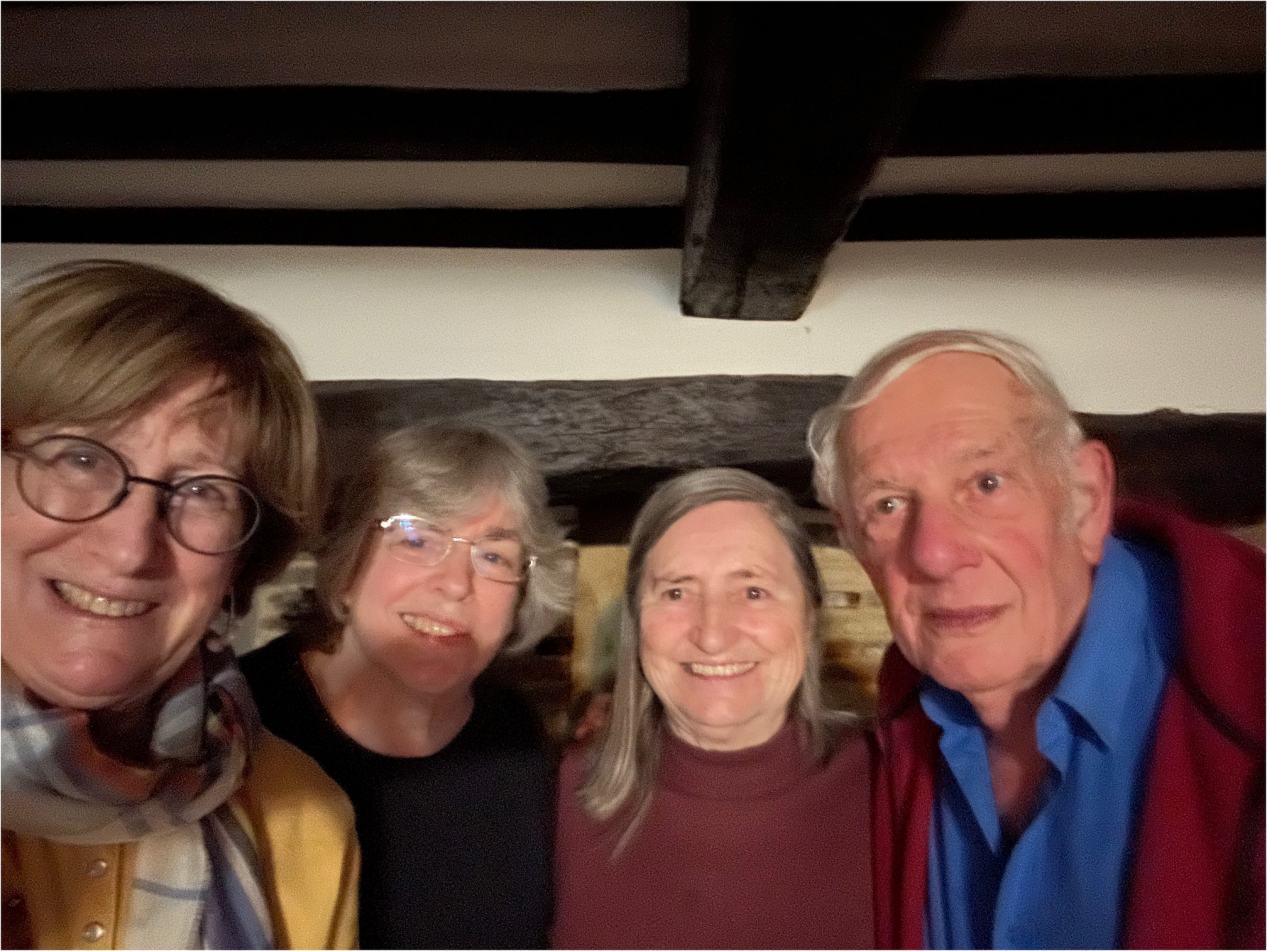
Selfie portrait of Frances Brodsky, Scottie Robinson, Barbara Pearse and Mark Bretscher (left to right), January 2026.

## Funding

F.M.B. was supported by UKRI MRC grant MR/X018377/1 and M.S.R. was supported by Wellcome Discovery Award 319762 while writing this article.

## Conflicts of Interest

The authors declare no conflicts of interest.

## Data Availability

Data sharing is not applicable to this article as no datasets were generated or analysed during the current study.
